# The Role of Plasma Membrane Pleiotropic Drug Resistance Transporters in the Killer Activity of *Debaryomyces hansenii* and *Wickerhamomyces anomalus* Toxins

**DOI:** 10.3390/toxins14030180

**Published:** 2022-02-28

**Authors:** Monika Czarnecka, Xymena Połomska, Cristina Restuccia, Barbara Żarowska

**Affiliations:** 1 Faculty of Biotechnology and Food Science, Wrocław University of Environmental and Life Sciences, ul. Chełmońskiego 37/41, 51-630 Wrocław, Poland; xymena.polomska@upwr.edu.pl (X.P.); barbara.zarowska@upwr.edu.pl (B.Ż.); 2 Department of Agriculture, Food and Environment (Di3A), University of Catania, via Santa Sofia 100, 95123 Catania, Italy; crestu@unict.it

**Keywords:** *Debaryomyces hansenii*, *Wickerhamomyces anomalus*, *Saccharomyces cerevisiae*, PDR transporters, killer toxin

## Abstract

The killer strains of *Debaryomyces hansenii* and *Wickerhamomyces anomalus* species secrete antimicrobial proteins called killer toxins which are active against selected fungal phytopathogens. In our research, we attempted to investigate the role of plasma membrane pleiotropic drug resistance (PDR) transporters (Pdr5p and Snq2p) in the mechanism of defense against killer toxins. *Saccharomyces cerevisiae* mutant strains with strengthened or weakened pleiotropic drug resistance due to increased or reduced number of mentioned PDR efflux pumps were tested for killer toxin susceptibility. The present study demonstrates the influence of the Snq2p efflux pump in immunity to *W.* *anomalus* BS91 killer toxin. It was also shown that the activity of killer toxins of *D. hansenii* AII4b, KI2a, MI1a and CBS767 strains is regulated by other transporters than those influencing *W. anomalus* killer toxin activity. In turn, this might be related to the functioning of the Pdr5p transporter and a complex cross-talk between several regulatory multidrug resistance networks. To the best of our knowledge, this is the first study that reports the involvement of PDR transporters in the cell membrane of susceptible microorganisms in resistance to killer yeasts’ toxins.

## 1. Introduction

Killer strains of the species *Debaryomyces hansenii* and *Wickerhamomyces anomalus* exhibit antagonistic activity against several yeast species and fungal phytopathogens. Thus, they are considered promising biocontrol agents in plant protection [1,2,3,4,5,6,7,8,9,10,11,12]. Antagonistic activity of *D. hansenii* and *W. anomalus* killer strains can be manifested through various mechanisms, including competition for nutrients and space, mycoparasitism, biofilm formation, induction of plants’ immune response to pathogens, and the secretion of antifungal substances, such as volatile organic compounds (VOCs), β-glucanases, and killer toxins [13,14,15,16,17,18]. The production of killer toxins, exhibiting antimicrobial activity, is a relatively common phenomenon in yeast. Strains with killer phenotypes produce killer toxins and are immune to their toxin. However, they may be susceptible to killer toxins produced by other killer yeasts [19,20]. The protein structure, specific receptors, and mode of action of killer toxins may differ significantly among yeast genera, species, and even strains. Most killer toxins exhibit a two-step killing mechanism. First, they bind to their specific receptors in the cell wall of susceptible microorganisms. Subsequently, toxins may exhibit killer mechanisms such as: blockage of DNA synthesis in the cell nucleus, cleavage of selected tRNAs, inhibition of β-1,3-glucan synthesis, disruption of electrochemical ion gradient across the plasma membrane, or enzymatic activity, leading to increased plasma membrane permeability with an eventual lethal effect [21,22,23,24]. 

The plasma membrane is essential for maintaining proper cell metabolism and osmotic pressure, for the exchange of genetic information and translocation of molecules across the plasma membrane. Compounds excreted from the cell are cellular metabolites, and xenobiotics, including antibiotics and antimicrobial substances. The main cellular elements responsible for the extrusion of numerous structurally unrelated molecules are the ATP-binding cassette (ABC) transporters, located in the cell membrane. These work as one-way pumps, thereby creating a multi-drug resistance (MDR) network. One of the main components of such a network in *S. cerevisiae* is the pleiotropic drug resistance (PDR) transporters system. PDR genes of *S. cerevisiae* share homology with mammalian MDR genes and some genes in other yeasts and fungi [25]. These homologies, with their ease of genome manipulation and simplicity of breeding, made *S. cerevisiae* a convenient and valuable tool for multidrug resistance research in medicine and agronomy [26].

**Figure 1:**
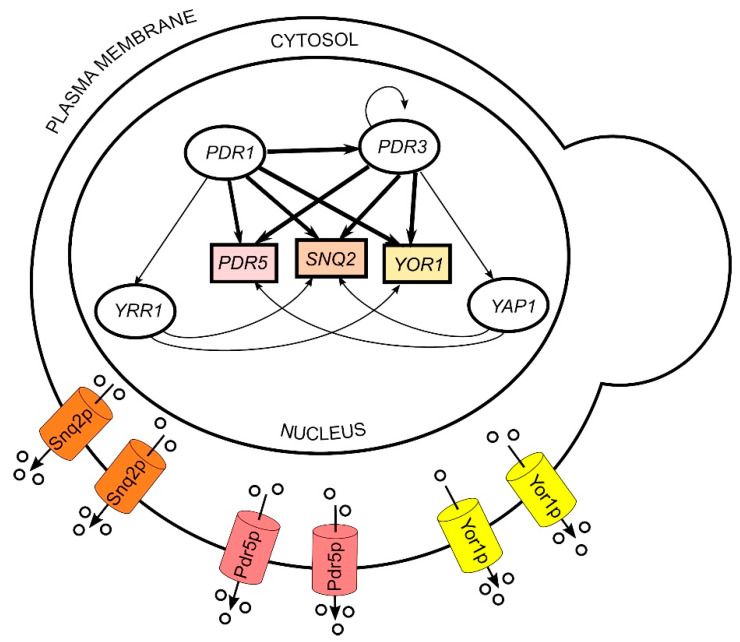
Positive regulation of *PDR5*, *SNQ2*, and *YOR1* genes encoding Pdr5p, Snq2p, and Yor1p efflux pumps, by prevailing transcription factors of PDR family: *PDR1* and *PDR3* and by *YAP1* and *YRR1*- transcription factors of interconnected with PDR drug resistance networks in *S. cerevisiae*.

There are three main regulatory networks involved in controlling the expression of multidrug resistance genes in *S. cerevisiae,* represented by different transcription factors: *PDR1* and *PDR3*, *YAP1* (yeast activator protein) and *YRR1* (yeast reveromycin resistance), from which *PDR1* and *PDR3* are the most characterized and studied [27]. *PDR5*, *SNQ2* (sensitivity to 4-*N*itroquinoline N-oxide) and *YOR1* (yeast oligomycin resistance) genes encoding Pdr5p, Snq2p, and Yor1p efflux pumps in the plasma membrane, are positively regulated mainly by *PDR1* and *PDR3* transcription factors. However, they may be also activated by *YAP1* or *YRR1* (Figure 1). These pumps are responsible for resistance to a wide spectrum of substrates, including molecules commonly used in the chemical treatment of fungal and bacterial infections [25,28,29]. *PDR1* and *PDR3* are homologous genes, at which point mutations lead to the overexpression of downstream target genes [30]. Single point mutations in the *PDR1* locus (*pdr1-2*, *pdr1-3*, *pdr1-6*, *pdr1-7*, *pdr1-8*) increase the expression of *PDR5*, *SNQ2*, *YOR1*, *PDR10*, *PDR15*, and *PDR16* genes, whereas mutations in *PDR3* (*pdr3-2* to *pdr3-10*) activate the expression of *PDR5*, *SNQ2*, *PDR15,* and *PDR3* genes, encoding efflux pumps in the cell membrane. Therefore, these point mutations enhance multidrug resistance in microorganisms. In turn, disruption, or deletion of *PDR1* and/or *PDR3* genes cause hypersensitivity, which is more pronounced in *PDR1* mutants [31,32,33,34,35]. The regulation of the PDR genes expression in microorganisms and plants may be also triggered by the exposure of cells to PDR-specific substrates [36,37,38,39].

So far, it has been established that β-glucans in the cell wall of sensitive microorganisms are the primary receptors for killer toxins of *D. hansenii* and *W. anomalus* [6,14,16,40,41,42,43]. However, little is known about the further effect of the killer toxins on the plasma membrane of sensitive microorganisms and potential defence systems. In this research, we attempt to establish whether plasma membrane components, such as PDR transporters, play a role in resistance to *D. hansenii* and *W. anomalus* killer toxins. In comparative studies, we analyzed the sensitivity of *S. cerevisiae* wild type strains and their isogenic mutants with increased or reduced pleiotropic drug resistance to *D. hansenii* and *W. anomalus* killer toxins. To the best of our knowledge, this is the first report considering the correlation between killer toxins activity and the function of plasma membrane transporters which contributes to the overall research on killer toxins and killer yeast antagonistic activity in biocontrol of pathogenic fungi.

## 2. Results

**Figure 2:**
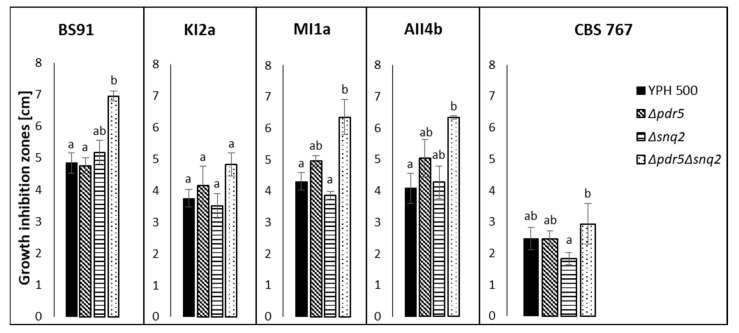
Growth inhibition zones of *S. cerevisiae* YPH500 wild type strain and its isogenic *Δpdr5*; *Δsnq2* and *Δpdr5Δsnq2* mutants in the presence of crude killer toxin preparations of *W. anomalus* BS91 and *D. hansenii* killer strains: KI2a, MI1a, AII4b, and CBS 767. Means and standard deviations of means are presented. The statistical significance of the differences between the means was tested separately for each killer toxin preparation (BS91, KI2a, MI1a, AII4b, and CBS 767) and marked with different letters. The study was performed with a Kruskal-Wallis one-way analysis of variance by ranks at *p* = 0.05. Means followed by different letters (a,b) are significantly different, while means marked by ab are not significantly different from groups a and b.

**Figure 3:**
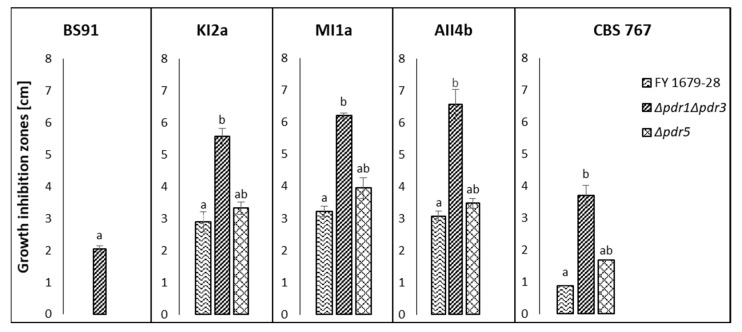
Growth inhibition zones of *S. cerevisiae* FY1679-28 wild type strain and its isogenic *Δpdr1Δpdr3* and *Δpdr5* mutants in the presence of crude killer toxin preparations of *W. anomalus* BS91 and *D. hansenii* killer strains: KI2a, MI1a, AII4b, and CBS 767. Means and standard deviations of means are presented. The statistical significance of the differences between the means was tested separately for each killer toxin preparation (BS91, KI2a, MI1a, AII4b, and CBS 767) and marked with different letters. The study was performed with a Kruskal-Wallis one-way analysis of variance by ranks at *p* = 0.05. Means followed by different letters (a,b) are significantly different, while means marked by ab are not significantly different from groups a and b.

**Figure 4:**
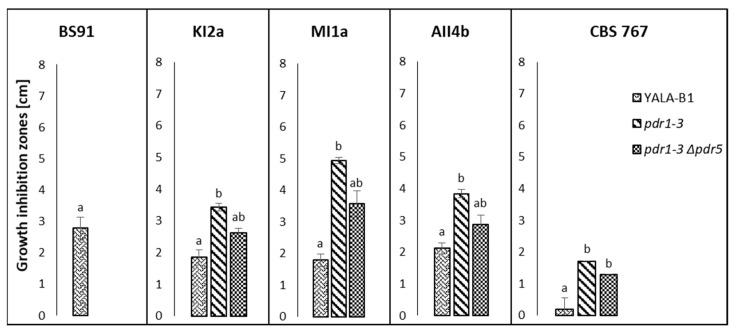
Growth inhibition zones of *S. cerevisiae* YALA-B1 wild type strain and its isogenic *pdr1-3* and *pdr1-3Δpdr5* mutants in the presence of crude killer toxin preparations of *W. anomalus* BS9 and *D. hansenii*: KI2a, MI1a, AII4b, and CBS 767. Means and standard deviations of means are presented. The statistical significance of the differences between the means was tested separately for each killer toxin preparation (BS91, KI2a, MI1a, AII4b, and CBS 767) and marked with different letters. The study was performed with a Kruskal-Wallis one-way analysis of variance by ranks at *p* = 0.05. Means followed by different letters (a,b) are significantly different, while means marked by ab are not significantly different from groups a and b.

PDR pumps are responsible for the translocation of small molecule compounds. However, in the present study, only crude killer toxins preparations exhibited the inhibitory effect on tested S. cerevisiae strains, whereas the small-molecule fractions (below 10 kDa), obtained from killer yeast cultures did not. Also, the post-culture fluids obtained from the culture of non-killer control strain D. hansenii CLIB 545 did not inhibit the growth of S. cerevisiae, thus confirming that the differences in the susceptibility of tested S. cerevisiae strains are toxin-dependent since CLIB 545 strain was grown under the same conditions as killer strains but the post culture medium did not contain killer toxins (Figure 2, Figure 3 and Figure 4). 

The growth of *S. cerevisiae* wild type strain YPH 500 and its isogenic mutants with deleted transporter genes *PDR5*, *SNQ2*, and *PDR5* together with *SNQ2* was inhibited by crude killer toxins of all tested killer yeast strains (AII4b, KI2a, MI1a, CBS 767, and BS91 at varying levels (Figure 2)). In general, the double deletant *(∆pdr5∆snq2*) was more susceptible to killer toxins than the parental YPH 500 strain or the single knock-outs *∆pdr5* and *∆snq2* (Figure 2). Deletion of *PDR5* resulted in higher sensitivity to killer toxins of *D. hansenii* AII4b, KI2a, and MI1a strains, and not to the toxin of *D. hansenii* CBS 767 and *W. anomalus* BS91 (Figure 2). Interestingly, *∆snq2* mutant was less sensitive to KI2a, MI1a, and CBS 767 in relation to isogenic wild type strain YPH 500, whereas in the presence of *W. anomalus* BS91 killer toxin, this strain was more sensitive than the wild type isogenic YPH 500 strain (Figure 2). 

Double deletion of genes encoding two main transcription factors *PDR1* and *PDR3* in *S. cerevisiae* FY1679-28 mutant resulted in significantly higher sensitivity to crude killer toxins of all tested killer *D. hansenii* yeast strains: AII4b, KI2a, MI1a, CBS 767, as compared to the wild type strain (Figure 3). Interestingly, the growth of wild-type FY 1679-28 strain and its isogenic *∆pdr5* mutant strain was not inhibited in the presence of *W. anomalus* BS9 killer toxin preparation, whereas double knock-out mutant (*∆pdr1∆pdr3*) appeared to be sensitive (Figure 3). 

Unexpectedly, the sensitivity of *S. cerevisiae* hyper-resistant *pdr1-3* mutant to all toxins of *D. hansenii*, was significantly higher, as compared to that of the wild type strain YALA-B1 (Figure 4). However, deletion of the *PDR5* transporter gene in a mutant strain with retained *pdr1-3* gain-of-function mutation resulted in a phenotype that was less sensitive to all killer toxins of *D. hansenii*, still more sensitive than wild-type strain YALA-B1. On the contrary, the *pdr1-3* mutant strain was completely resistant to the killer toxin of *W. anomalus* BS91. Also, there were no growth inhibition zones observed for *pdr1-3∆pdr5* isogenic mutant in the presence of BS91 toxin, whereas the growth of the control wild type YALA-B1 strain was inhibited by BS91 killer toxin (Figure 4). 

## 3. Discussion

*PDR5*, *SNQ2*, and *YOR1* genes, under prevailing control of *PDR1* and *PDR3* transcription factors, encode Pdr5p, Snq2p, and Yor1p efflux pumps in the cell membrane (Figure 1). These pumps are one of the most characterized transporters in the ABC family and exhibit extremely broad, sometimes overlapping, substrate specificity. The joint and compensatory action of these efflux pumps is responsible for the extrusion of various undesired small molecules and peptides from cells [25,44]. In turn, killer toxins derived from *D. hansenii* and *W. anomalus* are proteins of the molecular mass exceeding 10 kDa, which exhibit antagonistic activity towards sensitive microorganisms upon binding to their cell wall [1,12,14,17,41]. So far, there have been no reports that PDR transporters could transport large proteins across the cell membrane. In our experiments, only a large molecule fraction containing killer toxin exhibited an inhibitory effect on tested *S. cerevisiae*, while the small molecular fraction containing peptides and other small molecules did not. Therefore, it can be inferred that PDR pumps may play a role as plasma membrane components that are involved in resistance to killer toxins. However, these pumps may not necessarily work as a killer toxin extrusion system, and may instead modulate their activity.

**Figure 5:**
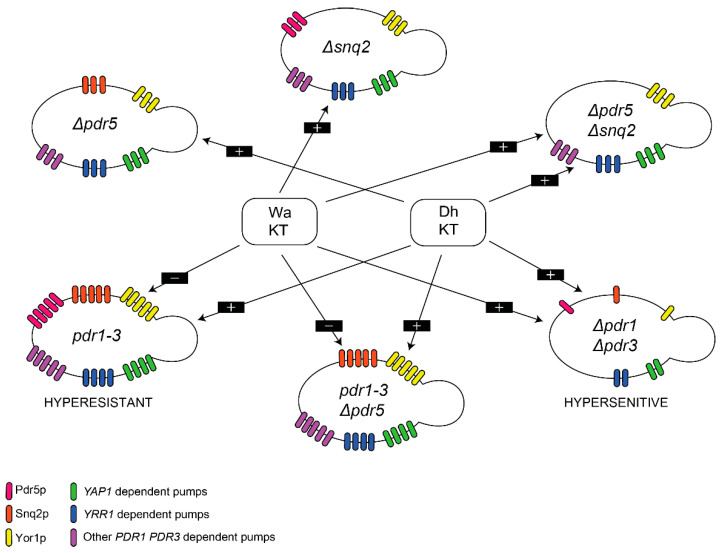
The schematic representation of the effect of type and quantity of transmembrane efflux pumps in *S. cerevisiae* PDR mutants on the sensitivity to *W. anomalus* (Wa) and *D. hansenii* (Dh) killer toxins (KT). The signs “+” and “−” in the black rectangle mean respectively: enhanced and decreased sensitivity to killer toxins.

In the present study, the role of PDR pumps in the killer toxin effect was more pronounced for *W. anomalus* BS91 killer toxin, as compared to *D. hansenii* killer toxins (Figure 5).

The activity of *W. anomalus* BS91 killer toxin towards *S. cerevisiae* mutants with a single deletion of *SNQ2* gene as well as deletion of both *SNQ2* and *PDR5* genes was enhanced as compared to the wild type strain YPH 500 and its single *PDR5* deletant suggesting the possible role of Snq2p in resistance to BS91 killer toxin. In turn, in *S. cerevisiae pdr1-3* hyper-resistant mutant, exhibiting enhanced expression of *PDR5*, *SNQ2* and *YOR1*, the activity of BS91 killer toxin was extinguished. The deletion of the *PDR5* gene in the *pdr1-3* genetic background did not result in regaining of susceptibility of cells to BS91 killer toxin suggesting that the Pdr5p transporter may not be involved in the inhibition of BS91 killer toxin activity, since the lack of this protein did not change the resistance of *pdr1-3* mutant to BS91 killer toxin. This was also shown in FY 1679-28 wild type strain and its *PDR5* single deletant. None of them was sensitive to BS91 toxin. A double deletant in *PDR1* and *PDR3* was significantly more sensitive to BS91 killer toxin than the isogenic wild type *S. cerevisiae* strain, again indicating the involvement of Snq2p and possibly Yor1p transporters in resistance to BS91 toxin (Figure 5). 

In turn, killer toxins of *D. hansenii* exhibited varying activity towards *S. cerevisiae* PDR mutants and the tendency in their performance differed from that of *W. anomalus* BS91 killer toxin. As was expected, double deletant in both major transcription factors *PDR1* and *PDR3* was significantly more susceptible to all killer toxins of *D. hansenii*. Also, the single deletion of *PDR5* and the double deletion of *PDR5* and *SNQ2* affected the activity of most *D. hansenii* killer toxins as compared to the wild type YPH 500 strain. Whereas, single deletion of *SNQ2* did not, suggesting the role of Pdr5p in resistance to *D. hansenii* killer toxins in susceptible cells. However, the gain-of-function *pdr1-3* mutant, bearing an armour of PDR efflux pumps including Pdr5p, Snq2p, and Yor1p in the plasma membrane, was unexpectedly more sensitive to all *D. hansenii* killer toxins than the wild type isogenic strain. Single deletion of the *PDR5* gene in the *pdr1-3* background resulted in a phenotype that was more sensitive to *D. hansenii* killer toxins than the wild type strain. However, it was still significantly less sensitive than the hyper-resistant *pdr1-3* mutant (Figure 5). It could be therefore inferred that the *D. hansenii* killer toxin may be either recognized and affected by Pdr5p transporter in the cell membrane with a regular density of PDR transporters, and not with an excess of efflux pumps as it is in the *pdr1-3* mutant, or that killer toxin activity may be influenced by other than tested efflux pumps in the cell membrane (possibly under the control of *YAP1* or *YRR1* transcription factors). Also, according to Kolaczkowska et al. [44], there exists a compensatory activation of one multidrug transporter upon disruption of genes encoding different multidrug transporters, which is accompanied by increased efflux of substrates specific for the activated ones. According to their studies, upon the disruption of the *PDR5* gene, the resistance to Yor1p- and Snq2p-specific substrates increases. Since *D. hansenii* killer toxin activity did not seem to depend on Snq2p in YPH 500 background, the role of Yor1p should be considered. Yor1p is, among others, the most important pump in the extrusion of Aureobasidin A– a cyclic depsipeptide produced by killer yeast *Aurobasidium pullulans*, toxic to several yeast species and filamentous fungi, such as *Botrytis cinerea*, *Monilinia* sp. and *Penicillium* sp. [45,46], towards which *D. hansenii* also exhibits antagonistic activity [16,47,48]. 

## 4. Conclusions

The present research, with the use of *S. cerevisiae* mutants in terms of PDR transporters responsible for the extrusion of substances of low molecular mass from cells, demonstrated that the killer effect may depend on the presence and density of PDR pumps in the plasma membrane of sensitive to killer toxin microorganisms, including Pdr5p and Snq2p (Figure 5). It also pointed out a new difference in killer phenomenon between killer strains of *D. hansenii* and *W. anomalus* species, where *W. anomalus* killer toxin was inhibited by the PDR transporters under the control of *PDR1* and *PDR3* transcription factors, whereas *D. hansenii* killer toxins activity was dependent on different transporters in plasma membrane than BS91 killer toxin, which could not be unambiguously defined. In previous studies, it was already proven that killer toxins derived from *D. hansenii* and *W. anomalus* exhibited varying antagonistic activity against several fungal plant pathogens through different mechanisms of action [15,16,18]. A comparative study of killer yeast strains of *D. hansenii* and *W. anomalus* species revealed their common feature, which is the recognition of β-glucans in the cell wall of the attacked microorganisms. It also pointed out a differentiating feature, which is the formation and release of antimicrobial volatile organic compounds, that was observed only in *W. anomalus*, and not in *D. hansenii* killer strains in combating pathogenic filamentous fungi [9,16]. This biocontrol mechanism of *W. anomalus* might be in turn attributed to the production of ethyl acetate [13,49] and 2-phenylethanol [50], from which the latter one may be ejected from *S. cerevisiae* cells by Pdr12p efflux pump, whose *PDR12* gene stays under the control of *WAR1* transcription factor [51,52]. In relation to biocontrol of phytopathogenic fungi, that contain MDR pumps in plasma membrane responsible for acquired resistance to chemical treatment of crops with azoles, strobilurins and succinate dehydrogenase inhibitors [53,54,55], this may elucidate the varying susceptibility of fungal pathogenic strains to killer toxins of *W. anomalus* and *D. hansenii*. As was shown in this study the inhibition of killer toxin produced by these yeast species relies on different PDR transporters. These findings shed new light on the perception of the mechanisms of the killer effect, understood as the antagonistic effect of killer yeasts against sensitive, often pathogenic yeast and microscopic fungi. Our novel findings constitute the foundations for research on the involvement of pleiotropic drug resistance efflux pumps of different families in the antagonistic effect of killer yeasts on sensitive microorganisms.

## 5. Materials and Methods

### 5.1. Yeast Strains

Killer strains of *D. hansenii* KI2a, MI1a and AII4b were isolated from blue-veined Rokpol cheese [56] and belong to the Culture Collection of the Department of Biotechnology and Food Microbiology at the Wroclaw University of Environmental and Life Sciences (Wroclaw, Poland). Another killer strain of *D. hansenii*, CBS 767 was obtained from CBS-KNAW Culture Collection. *W. anomalus* BS91 killer yeast was isolated from naturally fermented olive brine [57] and belongs to the Di3A Culture Collection (University of Catania, Italy). Killer phenotypes of these strains are presented in Table 1. *D. hansenii* CLIB 545, obtained from Centre International de Ressources Microbiennes (CIRM-Levures, http://www.inra.fr/cirmlevures, 16 August 2021), was used as a control non-killer, non-resistant to killer toxins strain (Table 1). *S. cerevisiae* yeast strains were chosen based on the characteristics of drug resistance associated with PDR transporters in their plasma membrane and their genotypes are presented in Table 2. YPH500 is an isogenic wild type strain to YKKB-13 strain with a single *PDR5* knock-out, YYM5 strain with a single *SNQ2* knock-out, and YYM3—with a double *PDR5* and *SNQ2* knock-outs. FY-1679-28C is an isogenic wild type strain to hyper-sensitive FY Δpdr1Δpdr3 double knock-out strain and a single *PDR5* knock-out strain FY-WT/Δpdr5-2. YALA-B1 is an isogenic strain to YALA-G4 hyper-resistant mutant (*pdr1-3*) and YZGA 278 hyper-resistant mutant (*pdr1-3*) with a single *PDR5* knock-out.

### 5.2. Killer Toxin Production

Yeasts were cultured on yeast peptone dextrose agar plates (YPDA; yeast extract, 10 g; peptone, 10 g; dextrose, 20 g; agar, 20 g per litre of distilled water) at 25 °C for 48 h. Then, the single colony biomass of each yeast strain was inoculated to 50 mL of YPD broth of pH adjusted to 4.5 with McIlvaine buffer and incubated either at 14 °C for 48 h (*D. hansenii* strains) or 25 °C (*W. anomalus*) for 24 h on a rotary shaker at 160 rpm to provide favourable conditions for killer toxin production. Yeast cells from liquid cultures were pelleted at 8000 g for 10 min. The supernatant was filtrated using a sterile syringe filter with a 0.22 µm pore size (Merck Millipore, Burlington, MA, USA, Milex-GP 33 mm, PES membrane). The obtained post-culture media served as crude toxin preparations. In addition, the small-molecule fractions (<10 kDa) were separated from the post-culture media using a filter with an appropriate cut-off point (Merck Millipore, Amicon^®^ Ultra-15, 10 kDa). Both crude killer toxin preparations and small-molecule fractions were used in the diffusion assay. 

### 5.3. Killer Toxin Diffusion Assay

The killer activity of *D. hansenii* and *W. anomalus* toxins was tested in agar diffusion bioassay according to the method described by Żarowska [12]. Twenty-five mL of YPDA medium, supplemented with 0.03% (*w*/*v*) methylene blue and 4% (*w*/*v*) of NaCl, was adjusted to pH 4.5 with McIlvaine buffer and used in the assay for the evaluation of killer toxin activity. Fifty µL of each tested sample (i) crude killer toxin preparation, (ii) small-molecule fraction (<10 kDa), was added into wells, sterilely cut in YPDA plates containing *S. cerevisiae* cells at a final concentration of 5 × 10^5^ per mL of the assay medium (Table 2). The zones of *S. cerevisiae* growth inhibition [cm] around wells were measured after 48 h of incubation at 20 °C. The experiment was repeated three times with three replicates for each tested sample. 

### 5.4. Statistical Analysis

In all repeated experiments, the arithmetic means were calculated and analyzed using Kruskal-Wallis one-way analysis of variance by ranks at *p* = 0.05. Post-culture media derived from the cultures of *D. hansenii* and *W. anomalus* were treated as individual preparations, the activity of which was tested against *S. cerevisiae* strains. The significant differences in growth inhibition zones between each *S. cerevisiae* wild type strain (YPH 500; FY-1679-28C and YALA-B1) and its isogenic PDR-mutants (YKKB-13, YYM 5, YMM3; FYΔpdr1Δpdr3, FY-WT/Δpdr5-2 and YALA-G4, YALA 278) were analyzed separately for each killer toxin preparation to demonstrate the role of PDR pumps on the toxic effect of all tested killer toxin preparations. The strength of the killer effect was not compared among killer strains. The results of mean killer activity of each preparation in relation to the tested *S. cerevisiae* strains, significantly different from each other, were grouped into homogenous groups a and b, while the results without statistically significant differences were assigned to the ab group, which was not significantly different from groups a or b. Data from experiments were analyzed using Statistica package software (Version 12; Statsoft Inc., Tulsa, OK, USA).

## Figures and Tables

**Table 1:** Killer phenotypes of *D. hansenii*, *W. anomalus,* and *S. cerevisiae* isogenic yeast strains used in this study.

Yeast Strain	Killer Phenotype
*D. hansenii* KI2a	K^+^R^+^
*D. hansenii* MI1a	K^+^R^+^
*D. hansenii* AII4b	K^+^R^+^
*D. hansenii* CBS 767	K^+^R^−^
*D. hansenii* CLIB 545	K^−^R^−^
*W. anomalus* BS91	K^+^R^+^
*S. cerevisiae* YPH 500	K^−^R^−D,−W^
*S. cerevisiae* FY 1679-28	K^−^R^−D,+W^
*S. cerevisiae* YALA-B1	K^−^R^−D,−W^

“K”—Killer; “R”—Resistant; “D”—*D. hansenii* killer toxins; “W”—*W. anomalus* killer toxin. K^+^R^+^—produces a killer toxin and is resistant to killer toxins; K^+^R^−^—produces a killer toxin and is sensitive to killer toxins produced by other killer strains; K^−^R^−^—does not produce a killer toxin and is sensitive to killer toxins.

**Table 2:** Genotype characterization of *S. cerevisiae* strains used in this study.

Strain *	Genotype	Reference
**YPH 500**	*MATα ura3-52 his3-Δ200 leu2-Δ1 trp1-Δ63 lys2-801^amber^ ade2-101^ochre^*	[34]
YKKB-13	*MATα ura3-52 his3-Δ200 leu2-Δ1 trp1-Δ63 lys2-801 ade2-101 pdr5Δ::TRP1*	[58]
YYM 5	*MATα ura3-52 his3-Δ200 leu2-Δ1 trp1-Δ63 lys2-801 ade2-101 snq2Δ::hisG*	[34]
YYM 3	*MATα ura3-52 his3-Δ200 leu2-Δ1 trp1-Δ63 lys2-801 ade2-101 pdr5Δ::TRP1 snq2Δ::hisG*	[35]
**FY-1679-28C**	*MATa ura3-53, leu2-∆1, trp1-∆63, his3∆200, GAL2^+^*	[30]
FY Δpdr1Δpdr3	*MATa ura3-53, leu2-∆1, trp1-∆63, his3∆200, GAL2^+^ pdr1-∆2::TRP1 pdr3-1∆::HIS3*	[30]
FY-WT/Δpdr5-2	*MATa ura3-53, leu2-∆1, trp1-∆63, his3∆200, GAL2^+^ pdr5*Δ*::URA3*	[59]
**YALA-B1**	*MAT*a *ura3-52 leu2-3,112 his3-11,115 trp1-1*	[35]
YALA-G4	*MAT*a *ura3-52 leu2-3,112 his3-11,115 trp1-1 pdr1-3*	[35]
YZGA 278	*MATa ura3-52, leu2-3,112 his3-11,115 trp1-1 pdr1-3 pdr5∆::hisG*	[59]

* The strains in bold are parental strains to the mutants listed below them in the table.

## Data Availability

The dataset used and/or analyzed during the current study are publicly available.
